# Risk factors for low-level viremia and virological failure in HIV patients in southwestern China who transitioned to second-line antiretroviral therapy after failure of first-line treatment: a retrospective cohort study

**DOI:** 10.3389/fpubh.2026.1932794

**Published:** 2026-10-08

**Authors:** Zongyu Nie, Yan Zheng, Liuying Yang, Shuanglian Ao, Hai Long, Peng Li

**Affiliations:** 1 School of Public Health, Guizhou University of Traditional Chinese Medicine, Guiyang, China; 2 Guiyang Public Health Clinical Center, Guiyang, China; 3 School of Geography and Environmental Science/Karst Research Institute, Guizhou Normal University, Guiyang, China

**Keywords:** COX proportional hazards regression model, HIV, LLV, second-line ART, VF

## Abstract

**Objective:**

To investigate factors associated with low-level viremia (LLV) and virological failure (VF) in HIV patients who transitioned to second-line antiretroviral therapy (ART) after first-line ART failure, aiming to inform management strategies for virological fluctuations among second-line ART patients in resource-limited settings.

**Methods:**

This retrospective cohort study included HIV patients who switched to second-line ART regimens between January 1, 2000, and January 1, 2026, due to first-line ART failure. Baseline demographic data (e.g., age at diagnosis, sex) and clinical indicators (e.g., time from diagnosis to ART initiation, baseline viral load, baseline CD_4_^+^ T lymphocyte count) at enrollment were collected. LLV and VF were defined as outcomes. Cox proportional hazards regression models were used to evaluate the associations between baseline characteristics/clinical indicators and LLV and VF.

**Results:**

A baseline CD_4_^+^ T lymphocyte count ≥350 was associated with a lower hazard of both LLV (aHR, 0.82; 95% CI: 0.60–0.88) and VF (aHR, 0.49; 95% CI: 0.29–0.76). HIV infection through homosexual transmission was associated with a higher hazard of LLV (aHR, 3.69; 95% CI: 1.48–9.23). Use of DTG + 3TC + TDF as a second-line regimen was associated with a higher hazard of both LLV (aHR, 2.25; 95% CI: 1.31–5.82) and VF (aHR, 2.23; 95% CI: 1.32–3.25). Co-infection with hepatitis B was associated with a higher hazard of LLV (aHR, 2.12; 95% CI: 1.13–6.23), whereas HCV seropositivity was associated with a higher hazard of both LLV (aHR, 3.01; 95% CI: 1.78–7.48) and VF (aHR, 2.79; 95% CI: 1.52–6.14). The M184V mutation was associated with a higher hazard of LLV (aHR, 6.35; 95% CI: 1.44–27.93). Both M184V (aHR, 4.76; 95% CI: 1.02–19.63) and K103N (aHR, 10.00; 95% CI: 4.12–24.30) mutations were associated with a higher hazard of VF.

**Conclusion:**

Lower baseline CD_4_^+^ T lymphocyte count, HIV infection through homosexual transmission, use of DTG + 3TC + TDF as a second-line regimen, hepatitis B co-infection or HCV seropositivity, and the presence of specific resistance-associated mutations were associated with a higher hazard of LLV and VF. Therefore, enhanced monitoring of resistance markers and immunological indicators is warranted among patients receiving second-line ART to facilitate timely clinical evaluation and treatment adjustment.

## 1 Introduction

In recent years, China has made significant progress towards achieving the UNAIDS 95-95-95 targets, with viral suppression (≤50 copies/mL) achieved in most HIV patients, reaching a high suppression rate of 91% (1). However, in the southwestern region of China, which bears a heavy burden of HIV disease and has relatively limited medical resources, HIV patients frequently face the risk of first-line ART failure due to delayed initiation of antiretroviral therapy (ART), a large proportion of migrant populations, and the high prevalence of hepatitis C and hepatitis B in the area (2). International guidelines recommend that following first-line ART failure, second-line antiretroviral therapy should consist of ritonavir-boosted protease inhibitors (PIs) or integrase strand transfer inhibitors (INSTIs), combined with two nucleoside reverse transcriptase inhibitors (NRTIs) (3). However, relevant literature indicates that patients on second-line ART have a 2.75 times higher risk of developing low-level viremia (LLV) compared to those on first-line ART (4). Moreover, persistent LLV can lead to the development of drug resistance mutations, increased release of inflammatory factors, heightened immune activation, and accelerated disease progression, ultimately resulting in virologic failure (VF) in HIV patients (5–8). Therefore, investigating the factors contributing to LLV and VF in second-line ART patients is crucial for optimizing treatment strategies and improving patient outcomes.

Currently, research on LLV and VF primarily focuses on initial antiretroviral treatment, with relatively few studies on the specific population of second-line ART patients. Most existing studies only suggest a higher risk of LLV and VF in second-line ART patients but do not systematically elucidate the specific factors contributing to these occurrences in this group. Furthermore, a systematic review presented at the 2023 International Antiviral Society conference indicated that the transmission risk associated with serum viral loads below 1,000 copies/mL is extremely low (9). However, other studies have found that in cases of mother-to-child transmission and injection drug use, if the blood viral load exceeds 12.7 copies/mL, merely 22.9 mL of blood transfusion could pose a risk of HIV transmission (10). Therefore, a comprehensive understanding of factors related to HIV viremia in second-line ART patients is of significant theoretical and practical importance for achieving the “Undetectable = Untransmittable” (U=U) goal and for managing virological fluctuations in clinical practice (11).

This study aims to investigate the factors associated with LLV and VF in HIV patients in Guizhou Province, Southwestern China, who initiated second-line ART regimens after first-line ART failure between January 1, 2000, and January 1, 2026, using collected follow-up data. Additionally, we evaluated clinical and epidemiological characteristics related to LLV and VF, with the aim of providing effective management strategies for virological fluctuations in second-line ART patients in clinical practice.

## 2 Methods

### 2.1 Study design and population

This study is a retrospective cohort study involving HIV patients in Guizhou Province, Southwest China, who were diagnosed between January 1, 2000, and January 1, 2026, and transitioned to second-line ART due to first-line treatment failure. The criteria for treatment failure were reviewed and confirmed by an expert panel during the data compilation phase, based on the 2023 “National Free Antiretroviral Treatment Manual for HIV/AIDS,” to ensure diagnostic consistency. Inclusion criteria for the study were as follows: patients aged 18 years or older who had received first-line ART for at least 6 months prior to switching to second-line therapy and achieved viral suppression (≤50 copies/mL) for at least six consecutive months after transitioning to second-line treatment. Patients entered the Cox risk-set at the end of this six-month sustained viral-suppression period. Only LLV and VF events occurring after this time point were defined as study outcomes. Participants presenting LLV or VF within this initial six-month window after second-line switch failed to meet the inclusion criteria and were excluded (see Figure 1).

**Figure 1:**
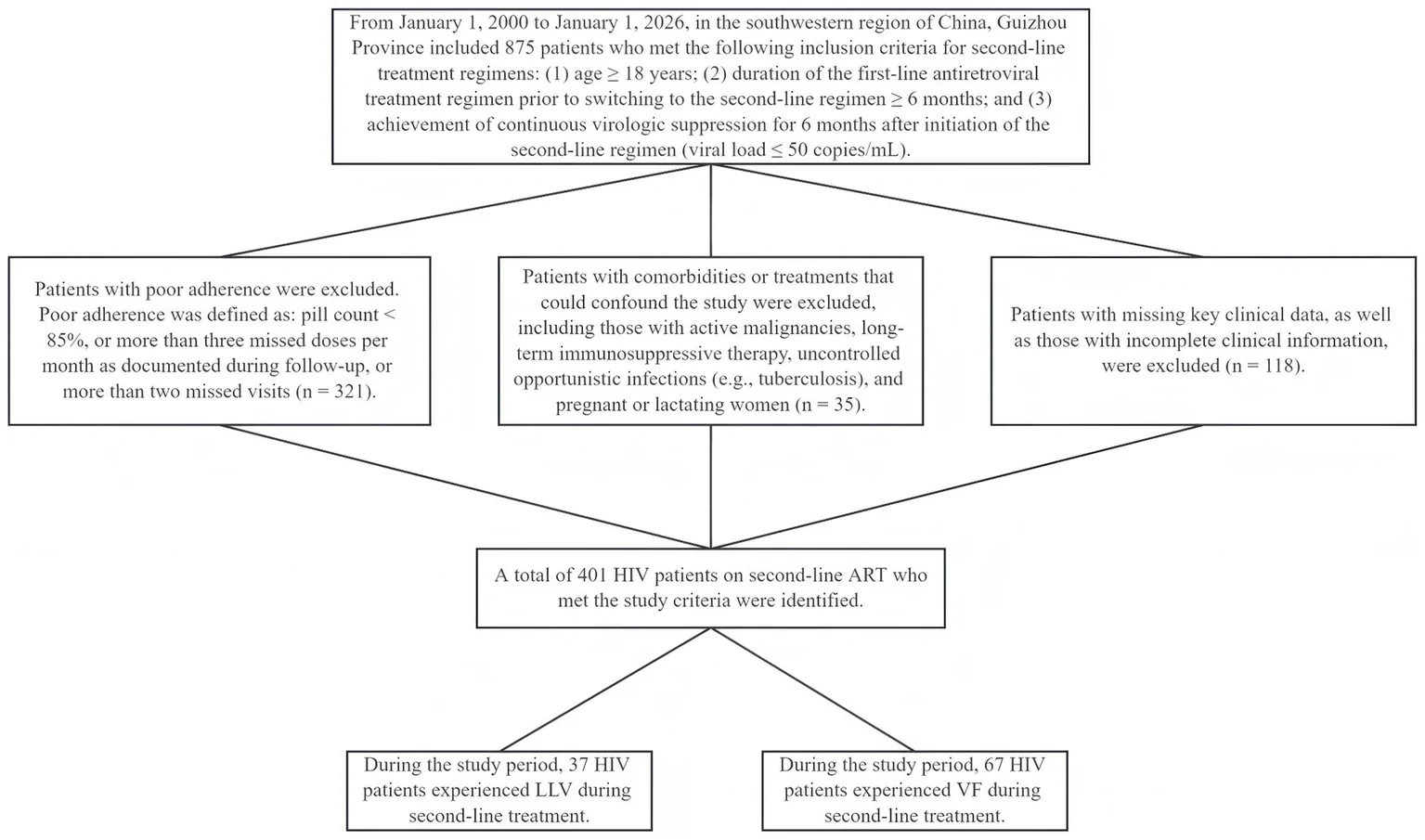
Flowchart for Exclusion Criteria of Second-Line ART HIV Patients.

In the study region, the selection of second-line ART regimens was individualized by clinicians based on each patient’s history of first-line treatment failure, prior antiretroviral drug exposure, baseline viral load, CD_4_^+^ T-lymphocyte count, clinical status, and the results of baseline genotypic resistance testing. All patients completed genotypic resistance testing before initiating second-line ART. Clinicians then selected appropriate second-line antiretroviral regimens in accordance with WHO second-line treatment principles, integrating the identified resistance-associated mutations into treatment decision-making.

### 2.2 Data collection

Data for this study were sourced from the standardized electronic medical record system of the Guizhou Provincial AIDS Diagnosis and Treatment Quality Control Center, which covers designated antiretroviral treatment hospitals across the province. Collected data included: demographic characteristics (sex, age at diagnosis, marital status, transmission route), clinical characteristics (WHO staging, baseline HIV viral load, baseline CD_4_^+^ T cell count, and drug resistance test results), and treatment and follow-up data (first-line/s-line ART regimens and switch dates, ART initiation date, and follow-up indicators every 6 months, such as viral load). Viral load was measured using the Roche COBAS TaqMan system (detection threshold 20 copies/mL). HBV coinfection was defined as positive baseline hepatitis B surface antigen (HBsAg). Patients with serological evidence of past HBV exposure (HBsAg-negative and anti-HBc-positive) were not classified as having HBV coinfection. HCV seropositivity was defined by positive baseline anti-HCV antibody. HCV-RNA testing was not systematically performed in this cohort; thus, we could not distinguish active HCV infection from resolved past HCV infection. CD_4_^+^ T lymphocyte counts were determined by flow cytometry, and drug resistance testing results were interpreted using the Stanford HIVDB algorithm. ART initiation time was categorized as rapid initiation (≤7 days), routine initiation (8–29 days), and delayed initiation (≥30 days). Data were independently extracted and verified by two researchers, and any discrepancies were resolved by reviewing the original medical records.

This study’s data were supported by the Guizhou Provincial AIDS Diagnosis and Treatment Quality Control Center and entrusted to the Ethics Committee of Guiyang Public Health Treatment Center for ethical review. All experts involved in the validation signed informed consent forms. Due to the retrospective nature of the study, the Ethics Committee of Guiyang Public Health Clinical Center waived the requirement for patient informed consent (Approval No.: 2025-73). This study strictly adhered to the ethical standards of the Declaration of Helsinki.

### 2.3 Outcome measures

This study aimed to evaluate factors associated with LLV and VF among people living with HIV who switched to second-line ART following failure of first-line treatment. In the primary analysis, LLV was defined as plasma HIV-1 RNA levels of 51–999 copies/mL on two consecutive measurements taken at least 1 month apart, with no ART regimen modification. VF was defined as plasma HIV-1 RNA levels ≥1,000 copies/mL on two consecutive measurements taken at least 1 month apart, with no ART regimen modification.

In the sensitivity analysis, participants were classified based on the first virological episode that fulfilled the prespecified LLV or VF criteria during follow-up. Specifically, each episode required two consecutive viral load measurements meeting the corresponding threshold, taken at least 1 month apart, with no ART regimen modification. If a participant first met the complete definition of LLV and later subsequently met the definition of VF, the participant was classified in the LLV group; conversely, if a participant first met the complete definition of VF, the participant was classified in the VF group. Given the low frequency of recurrent episodes of LLV and VF, the analyses primarily focused on factors associated with the first episode of LLV or VF during subsequent follow-up among participants who achieved sustained viral suppression for up to 6 months after initiating second-line ART.

### 2.4 Statistical analysis

Demographic data and clinical characteristics were described using medians and quartiles, and categorical variables were presented as frequencies and percentages. Between-group differences were statistically analyzed using the *χ*^2^ or Fisher exact test. Cox proportional hazards models were used to evaluate factors potentially associated with low-level viremia and virological rebound, and all potential confounding variables were included in the multivariable Cox proportional hazards regression analysis. In addition, to avoid multicollinearity, the study used a complete case approach, and any participant visits with missing data were excluded from the multivariable model. All statistical analyses were performed in R software (version 4.3.1), all tests were two-sided, and statistical significance was set at *p* < 0.05.

## 3 Results

### 3.1 Study population

Between January 1, 2000 and January 1, 2026, a total of 401 people living with HIV in Southwest China were switched to second-line ART after failure of first-line ART. This population was predominantly male, with a median age at diagnosis of 36.4 years (interquartile range: 26.9–47.6 years). Participants with LLV or VF did not show significant differences in age at diagnosis. However, among patients with VF, most had a time from diagnosis to treatment of ≥30 days, suggesting that delayed treatment may be associated with virological rebound. In addition, most patients with LLV and patients with VF acquired HIV through heterosexual transmission. In the LLV group, the proportion of patients with CD_4_^+^ T lymphocyte counts of 0–200 was higher than in the other groups. Similarly, in the VF group, the proportion of patients with CD_4_^+^ T lymphocyte counts of 0–200 was also significantly higher (See Table 1).

**Table 1:** Participant characteristics.

Characteristic	Overal	NO LLV	LLV	*p*	No VF	VF	*p*
Diagnosis age				0.438			0.432
18–29	118 (29.43)	102 (28.02)	16 (43.24)		92 (27.54)	26 (38.81)	
30–49	180 (44.89)	167 (45.88)	13 (35.14)		152 (45.51)	28 (41.79)	
≥50	103 (25.68)	95 (26.10)	8 (21.62)		90 (26.95)	13 (19.40)	
Gender				0.197			0.951
Male	317 (79.05)	292 (80.22)	25 (67.57)		265 (79.34)	52 (77.61)	
Female	84 (20.95)	72 (19.78)	12 (32.43)		69 (20.66)	15 (22.39)	
Time to initiate ART				0.996			0.011
≤7	141 (35.16)	127 (34.89)	14 (37.84)		130 (38.92)	11 (16.42)	
8–29	96 (23.94)	87 (23.90)	9 (24.32)		73 (21.86)	23 (34.33)	
≥30	164 (40.90)	150 (41.21)	14 (37.84)		131 (39.22)	33 (49.25)	
Baseline viral load				0.848			0.997
≤10,000	211 (52.63)	189 (51.92)	22 (59.46)		176 (52.69)	35 (52.24)	
10,000–99,999	83 (20.69)	78 (21.43)	5 (13.51)		70 (20.96)	13 (19.40)	
≥100,000	107 (26.68)	97 (26.65)	10 (27.03)		88 (26.35)	19 (28.36)	
Viral load before second-line treatment				0.975			0.067
≤10,000	260 (64.84)	237 (65.11)	23 (62.16)		227 (67.96)	33 (49.25)	
10,000–99,999	100 (24.94)	91 (25.00)	9 (24.33)		75 (22.46)	25 (37.31)	
≥100,000	41 (10.22)	36 (9.89)	5 (13.51)		32 (9.58)	9 (13.44)	
Baseline CD4 CellCount				0.183			0.737
0–200	266 (66.33)	248 (68.13)	18 (48.65)		217 (64.97)	49 (73.13)	
201–350	84 (20.95)	71 (19.51)	13 (35.14)		74 (22.16)	10 (14.93)	
≥351	51 (12.72)	45 (12.36)	6 (16.21)		43 (12.87)	8 (11.94)	
CD4 cell count before second-line treatment				0.859			0.031
0–200	216 (53.87)	199 (54.67)	17 (45.95)		190 (56.89)	26 (38.81)	
201–350	92 (22.94)	81 (22.25)	11 (29.73)		67 (20.06)	25 (37.31)	
≥351	93 (23.19)	84 (23.08)	9 (24.32)		77 (23.05)	16 (23.88)	
Marital status				0.731			0.903
Single	156 (38.90)	145 (39.84)	11 (29.73)		133 (39.82)	23 (34.33)	
Married	187 (46.63)	166 (45.60)	21 (56.76)		152 (45.51)	35 (52.24)	
Divorced or Widowed	43 (10.72)	38 (10.44)	5 (13.51)		35 (10.48)	8 (11.94)	
Other	15 (3.75)	15 (4.12)	0 (0)		14 (4.19)	1 (1.49)	
Routes of infection				0.043			<0.001
Heterosexual transmission	211 (52.62)	183 (50.27)	28 (75.68)		162 (48.50)	49 (73.14)	
Homosexual transmission	144 (35.91)	139 (38.19)	5 (13.51)		130 (38.92)	14 (20.89)	
Others	46 (11.47)	42 (11.54)	4 (10.81)		42 (12.58)	4 (5.97)	
WHO clinical staging				<0.001			0.744
1	174 (43.39)	163 (44.78)	11 (29.73)		141 (42.22)	33 (49.25)	
2	35 (8.73)	30 (8.24)	5 (13.51)		29 (8.68)	6 (8.96)	
3	40 (9.98)	35 (9.62)	5 (13.51)		32 (9.58)	8 (11.94)	
4	152 (37.90)	136 (37.36)	16 (43.25)		132 (39.52)	20 (29.85)	
Second-line treatment regimen				0.531			0.407
LPV/r + 3TC + AZT	167 (41.65)	150 (41.21)	17 (45.95)		141 (42.22)	26 (38.81)	
LPV/r + 3TC + TDF	172 (42.89)	154 (42.31)	18 (48.65)		137 (41.02)	35 (52.24)	
DTG + 3TC + TDF	62 (15.46)	60 (16.48)	2 (5.40)		56 (16.76)	6 (8.95)	
History of hepatitis Bb				0.974			0.005
No	353 (88.03)	320 (87.91)	33 (89.19)		291 (87.13)	62 (92.54)	
Yes	48 (19.97)	44 (12.09)	4 (10.81)		43 (12.87)	5 (7.46)	
HCV seropositivity				0.379			0.570
No	378 (94.26)	345 (94.78)	33 (89.19)		313 (93.71)	65 (97.01)	
Yes	23 (5.74)	19 (5.22)	4 (10.81)		21 (6.29)	2 (2.99)	
Resistance-associated mutations							0.008
No	314 (78.30)	300 (82.42)	14 (37.84)	<0.001	266 (79.64)	48 (71.64)	
M184V	23 (5.74)	4 (1.09)	19 (51.35)		21 (6.29)	2 (2.99)	
K103N	20 (4.99)	19 (5.22)	1 (2.70)		10 (2.99)	10 (14.93)	
Multiple TAMs Co-mutations	26 (6.48)	23 (6.32)	3 (8.11)		24 (7.19)	2 (2.99)	
Other mutation sites	18 (4.49)	18 (4.95)	0 (0)		13 (3.89)	5 (7.45)	

LLV, Low Viral Load; VF, Viral Failure; ART, antiretroviral therapy; HIV, human immunodeficiency virus; Using X^2^ or Fisher’s exact test to compare the characteristics of participants with and without LLV and VF, statistical significance comparisons (*p* < 0.05) are highlighted in bold.

### 3.2 Factors related to LLV

In the multivariable model including 401 HIV patients, when baseline CD_4_^+^ T lymphocyte count was ≥350 (aHR, 0.82; 95% CI: 0.60–0.88), LLV occurred at a lower hazard compared with patients whose baseline CD_4_^+^ T lymphocyte count was 201–350. Thus, a higher baseline CD_4_^+^ T lymphocyte count was associated with a lower hazard of LLV. In addition, the mode of HIV infection was associated with the incidence of LLV. HIV acquisition via homosexual transmission was associated with a higher hazard of LLV compared with other routes of infection (reference group) (aHR, 3.69; 95% CI: 1.48–9.23). DTG + 3TC + TDF was associated with a higher hazard of LLV relative to the reference regimen LPV/r + 3TC + AZT (aHR, 2.25; 95% CI: 1.31–5.82). A third second-line regimen included in the analysis was LPV/r + 3TC + TDF. In the multivariable Cox regression model, compared with patients without HBV coinfection, hepatitis B coinfection (aHR = 2.12; 95% CI: 1.13–6.23) was associated with a higher hazard of LLV, and compared with patients without HCV seropositivity, HCV seropositivity (aHR = 3.01; 95% CI: 1.78–7.48) was also associated with a higher hazard of LLV. Regarding drug resistance, specific resistance sites showed significant associations with LLV. Specifically, patients harboring the M184V mutation (aHR = 6.35; 95% CI: 1.44–27.93) had a significantly higher hazard of LLV (see Table 2; Figure 2). Overall, these findings suggest that resistance-associated mutations (RAMs) may be associated with LLV, and clinical practice should consider enhanced monitoring and management of relevant RAMs.

**Table 2:** Factors associated with the first LLV.

Characteristic	Unadjusted HR (95% CI)	Adjusted HR (95% CI)
Diagnosis age		
18–29	Reference	
30–49	**0.43 (0.18–0.97)**	0.49 (0.21–1.5)
≥50	0.77 (0.31–1.90)	0.84 (0.32–2.17)
Gender		
Female	Reference	
Male	0.71 (0.28–1.74)	0.67 (0.26–1.72)
Time to initiate ART		
≤7	Reference	
8–29	0.58 (0.21–1.65)	0.59 (0.20–1.68)
≥30	1.09 (0.50–2.36)	1.14 (0.50–2.61)
Baseline viral load		
≤10,000		
10,000–99,999	0.64 (0.27–5.67)	0.75 (0.27–2.13)
≥100,000	0.78 (0.34–1.79)	0.79 (0.32–1.93)
Viral load before second-line treatment		
≤10,000	Reference	
10,000–99,999	0.73 (0.31–1.73)	0.83 (0.34–2.05)
≥100,000	1.09 (0.37–3.22)	1.40 (0.43–4.49)
Baseline CD4 CellCount		
0–200	Reference	
201–350	**3.69 (1.71–8.01)**	**3.97 (1.69–9.33)**
≥351	**0.84 (0.62–0.91)**	**0.82 (0.60–0.88)**
CD4 cell count before second-line treatment		
0–200		
201–350	0.92 (0.38–2.17)	0.84 (0.32–2.19)
≥351	0.43 (0.17–1.06)	0.44 (0.17–1.09)
Marital status		
Other	Reference	
Single	0.82 (0.35–1.91)	1.03 (0.39–2.65)
Married	1.49 (0.58–3.88)	1.91 (0.66–5.53)
Divorced or Widowed	1.37 (0.17–10.99)	0.69 (0.07–6.81)
Routes of infection		
Others	Reference	
Heterosexual transmission	0.86 (0.12–6.47)	1.05 (0.05–3.87)
Homosexual transmission	1.96 (0.90–4.24)	**3.69 (1.48–9.23)**
WHO clinical staging		
1	Reference	
2	0.72 (0.23–2.23)	0.74 (0.23–2.33)
3	1.48 (0.53–4.18)	1.40 (0.49–3.98)
4	0.63 (0.25–1.57)	0.44 (0.16–1.18)
Second-line treatment regimen		
LPV/r + 3TC + AZT	Reference	
LPV/r + 3TC + TDF	0.90 (0.43–1.89)	0.90 (0.43–1.90)
DTG + 3TC + TDF	**2.12 (1.29–5.60)**	**2.25 (1.31–5.82)**
History of hepatitis Bb		
No	Reference	
Yes	**2.06 (1.04–5.20)**	**2.12 (1.13–6.23)**
HCV seropositivity		
No	Reference	
Yes	**2.78 (1.12–5.38)**	**3.01 (1.78–7.48)**
Resistance-associated mutations		
No	Reference	
M184V	**5.72 (1.32–24.79)**	**6.35 (1.44–27.93)**
K103N	1.10 (0.11–1.20)	1.10 (0.13–1.20)
Multiple TAMs co-mutations	2.38 (0.56–10.09)	2.14 (0.49–9.26)
Other mutation sites	1.25 (0.17–9.23)	1.33 (0.18–10.02)

The Cox proportional hazards model was used to calculate the unadjusted and adjusted hazard ratios (HR) and their 95% confidence intervals (CI), with statistically significant HRs (*p* < 0.05) displayed in bold.

**Figure 2:**
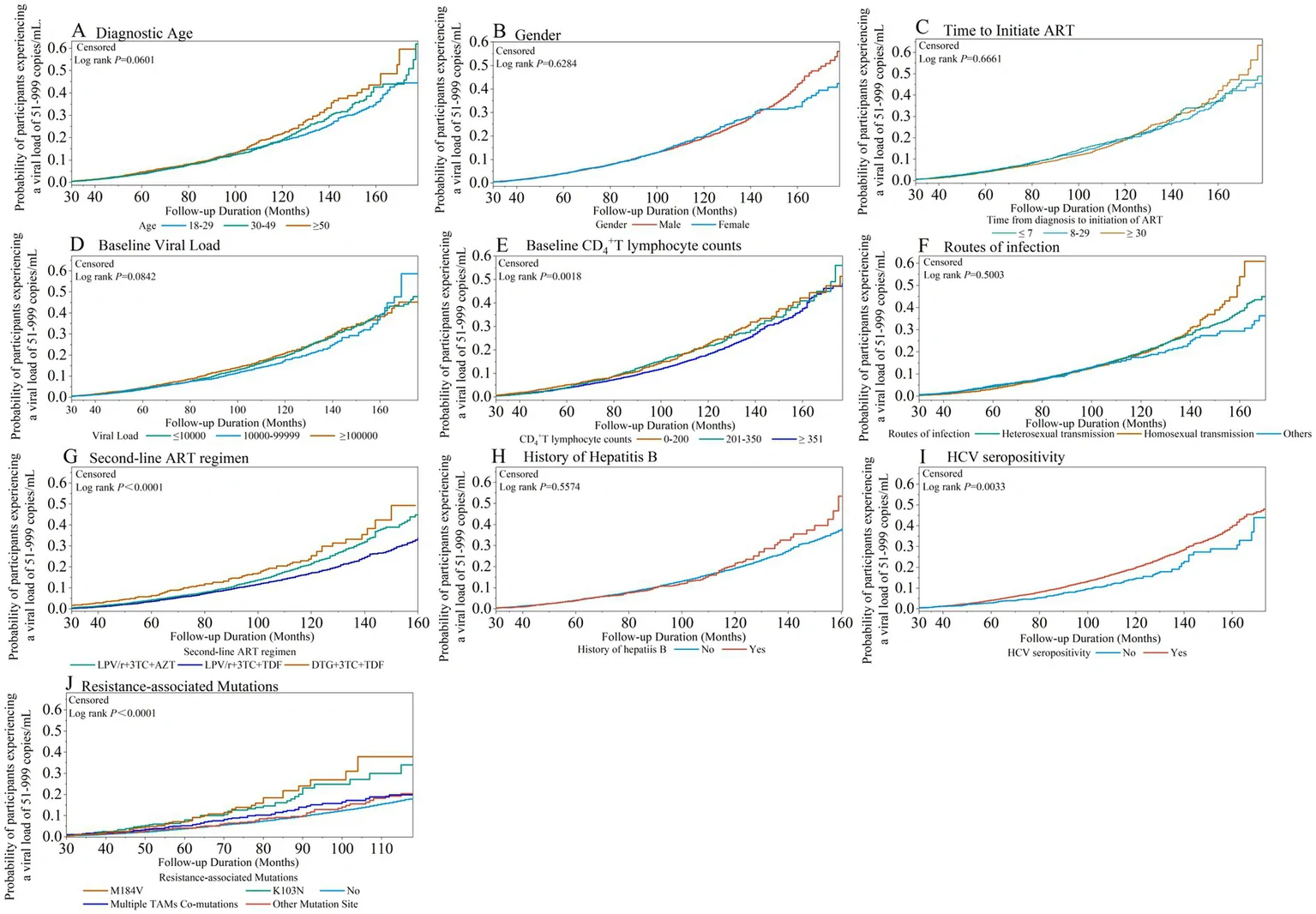
Kaplan–Meier Survival Curves for Factors Influencing Time to First Low-Level Viremia (LLV) in HIV Patients, Survival Curves Based on: **(A)** Age **(B)** Gender **(C)** Time to ART Initiation **(D)** Baseline Viral Load **(E)** Baseline CD4^+^ T Lymphocyte Counts **(F)** Routes of Infection **(G)** Second-line ART Regimen **(H)** Hepatitis B History **(I)** HCV seropositivity **(J)** Resistance-associated Mutations.

### 3.3 Factors related to VF

This study assessed factors associated with VF by constructing a Cox proportional hazards model, and found that baseline CD_4_^+^ T lymphocyte count affected the risk of VF in HIV patients. The study found that patients with a baseline CD_4_^+^ T lymphocyte count ≥351 (aHR = 0.49; 95% CI: 0.29–0.76) had a lower risk of VF than other groups, suggesting that the lower the immune function, the more difficult virological control may be. The second-line ART regimen used by patients was also associated with the risk of VF, patients using DTG + 3TC + TDF (aHR = 2.23; 95% CI: 1.32–3.25) as the treatment regimen had a higher risk of VF than patients in other groups. In addition, HCV seropositivity (aHR = 2.79; 95% CI: 1.52–6.14) was associated with a higher hazard of VF. RAMs were associated with VF. Compared with the reference group (without these RAMs), patients carrying the K103N mutation (aHR = 10.00; 95% CI: 4.12–24.30) and patients with the M184V mutation (aHR = 4.76; 95% CI: 1.02–19.63) had a higher adjusted hazard of VF (see Table 3; Figure 3).

**Table 3:** Factors associated with the first VF.

Characteristic	Unadjusted HR (95% CI)	Adjusted HR (95% CI)
Diagnosis age		
18–29	Reference	
30–49	0.81 (0.41–1.58)	0.74 (0.37–1.47)
≥50	1.01 (0.47–2.17)	1.02 (0.47–2.20)
Gender		
Female	Reference	
Male	1.07 (0.55–2.08)	1.19 (0.58–2.46)
Time to initiate ART		
≤7	Reference	
8–29	0.92 (0.45–1.89)	0.95 (0.46–1.98)
≥30	1.47 (0.73–2.98)	1.58 (0.77–3.28)
Baseline viral load		
≤10,000	Reference	
10,000–99,999	0.61 (0.28–1.33)	0.62 (0.28–1.39)
≥100,000	0.58 (0.28–1.19)	0.61 (0.29–1.30)
Viral load before second-line treatment		
≤10,000	Reference	
10,000–99,999	1.17 (0.63–2.15)	1.24 (0.66–2.32)
≥100,000	0.32 (0.08–1.34)	0.44 (0.10–1.94)
Baseline CD4 cell count		
0–200	Reference	
201–350	0.79 (0.39–1.63)	0.86 (0.41–1.81)
≥351	**0.59 (0.33–0.82)**	**0.49 (0.29–0.76)**
CD4 cell count before second-line treatment		
0–200	Reference	
201–350	1.9 (0.97–3.73)	1.63 (0.78–3.41)
≥351	0.72 (0.33–1.57)	0.68 (0.31–1.49)
Marital status		
Other	Reference	
Single	1.08 (0.14–8.40)	3.40 (0.38–30.39)
Married	1.05 (0.14–7.88)	4.92 (0.54–44.89)
Divorced or widowed	1.56 (0.19–12.22)	7.54 (0.79–71.97)
Routes of infection		
Others	Reference	
Heterosexual transmission	**0.37 (0.14–0.96)**	0.38 (0.13–1.16)
Homosexual transmission	0.59 (0.20–1.72)	0.62 (0.15–2.61)
WHO clinical staging		
1	Reference	
2	0.52 (0.19–1.7)	0.60 (0.22–1.64)
3	0.92 (0.35–2.45)	0.98 (0.36–2.65)
4	0.79 (0.40–1.55)	0.76 (0.36–1.61)
Second-line treatment regimen		
LPV/r + 3TC + AZT	Reference	
LPV/r + 3TC + TDF	1.14 (0.26–4.99)	1.06 (0.24–4.66)
DTG + 3TC + TDF	**2.12 (1.21–3.17)**	**2.23 (1.32–3.25)**
History of hepatitis BB		
No		
Yes	1.79 (0.75–4.27)	1.95 (0.77–4.94)
HCV seropositivity		
No	Reference	
Yes	**2.27 (1.39–4.12)**	**2.79 (1.52–6.14)**
Resistance-associated mutations		
No	Reference	
M184V	**4.48 (1.05–19.02)**	**4.76 (1.02–19.63)**
K103N	**9.86 (4.06–18.36)**	**10.00 (4.12–24.30)**
Multiple TAMs Co-mutations	0.76 (0.22–4.31)	0.86 (0.12–6.31)
Other mutation sites	1.66 (0.23–5.63)	1.87 (0.45–7.83)

Bold values represent statistically significant associations (*p* < 0.05).

**Figure 3:**
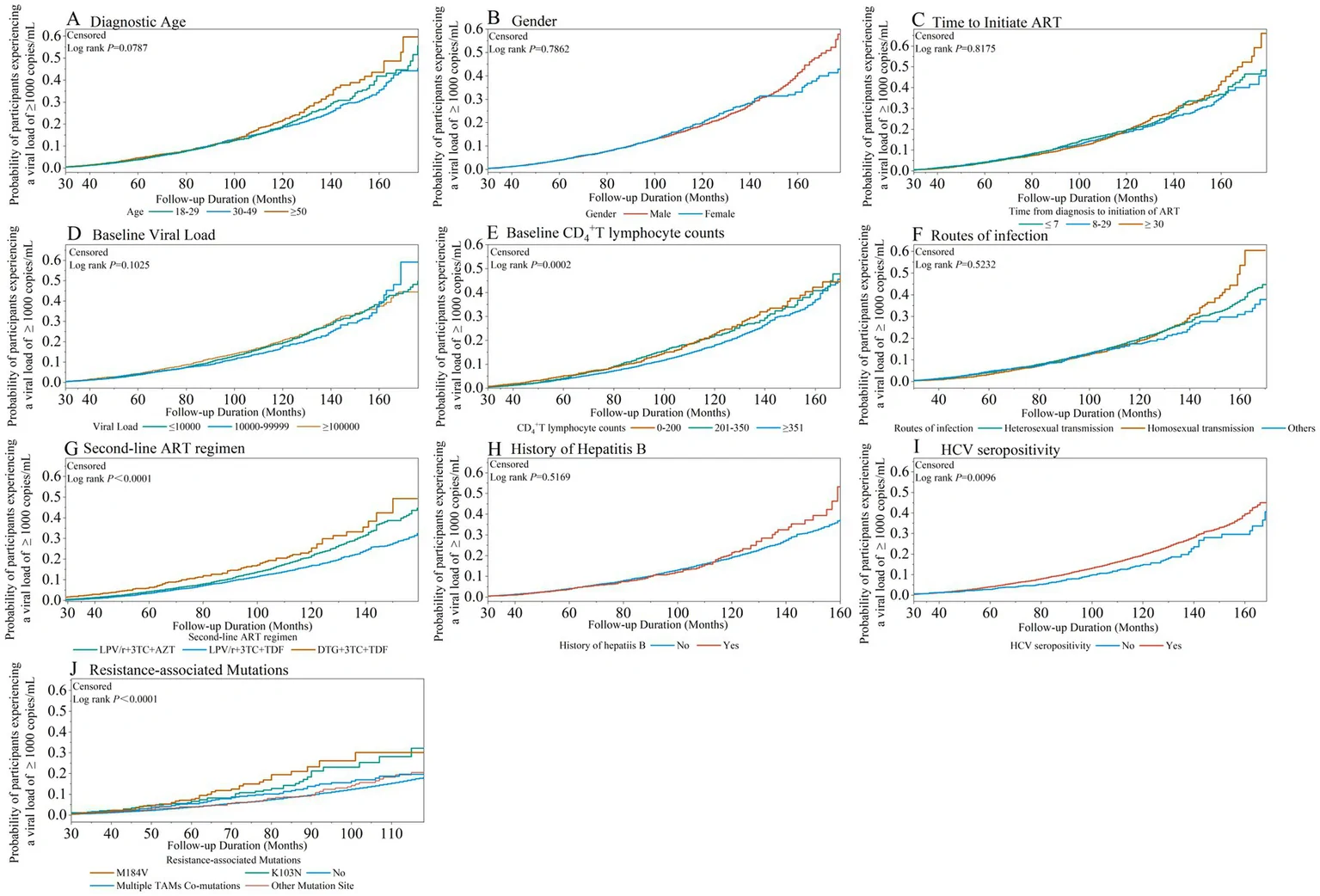
Kaplan–Meier Survival Curves for Factors Influencing Time to First Virologic Failure (VF) in HIV Patients, Survival Curves Based on: **(A)** Age **(B)** Gender **(C)** Time to ART Initiation **(D)** Baseline Viral Load **(E)** Baseline CD4^+^ T Lymphocyte Counts **(F)** Routes of Infection **(G)** Second-line ART Regimen **(H)** Hepatitis B History **(I)** HCV seropositivity **(J)** Resistance-associated Mutations.

## 4 Discussion

This retrospective cohort study included 401 patients who switched to a second-line regimen after failing first-line antiretroviral therapy, aiming to investigate risk factors for the occurrence of LLV and VF during second-line treatment. The study revealed that a higher baseline CD_4_^+^ T lymphocyte count was a protective factor against LLV. Factors associated with an increased risk of LLV included HIV infection acquired through homosexual transmission, using DTG + 3TC + TDF as the second-line regimen, hepatitis B co-infection or HCV seropositivity, and carrying the M184V mutation. Similarly, a higher baseline CD_4_^+^ T lymphocyte count was also a protective factor against VF, while the second-line regimen DTG + 3TC + TDF, hepatitis B co-infection or HCV seropositivity, and carrying the K103N and M184V mutations were associated with an increased risk of VF.

A study reported that young individuals with HIV are more likely to experience stigma during ART, which can worsen medication adherence, compromise treatment outcomes, and increase the risk of developing LLV and VF (12). However, this study did not find an association between age and either LLV or VF. This might be related to China’s “Four Frees and One Care” policy, which, by waiving some HIV testing and treatment costs and enhancing public education on HIV/AIDS knowledge, effectively eliminates societal misunderstandings about virus transmission routes and infected individuals. This encourages more young patients to seek medical attention promptly and take medication as prescribed (13). Although the implementation of this policy makes it easier for HIV patients to access healthcare services, older patients often face issues such as declining immune function, which can affect the efficacy of antiretroviral therapy. This effect can persist into second-line treatment, increasing the risk of LLV and VF.

Multiple studies have found that initiating ART during the acute or early stages of HIV infection (within the first few months of initial infection) has benefits for immune recovery in people living with HIV (14–21). Related research also indicates that early ART initiation can suppress viral fluctuations for up to 2 years (22). However, in this study, the impact of ART initiation time on LLV and VF in HIV patients was not found to be particularly strong. It is speculated that the virological and immunological advantages of early ART initiation primarily manifest during the first-line treatment phase. This study, however, mainly involved patients who had failed first-line treatment and transitioned to second-line therapy. The majority of these patients exhibited a certain degree of drug resistance, and the failure of first-line treatment weakened the protective effect brought by early ART initiation. Furthermore, the suppressive effect of early ART intervention on viral fluctuations might be time-limited, with its benefits gradually diminishing as the course of infection progresses.

Furthermore, in this study, a higher baseline viral load was not strongly associated with the risk of LLV and VF during second-line ART. However, a higher baseline CD_4_^+^ T lymphocyte count was a protective factor for both LLV and VF, which is consistent with previous research (23, 24). Before ART initiation, the formation of the viral reservoir leads to CD_4_^+^ T lymphocyte depletion and impaired immune function. Coupled with the potential development of resistance to certain antiviral drugs in patients who failed first-line treatment, this results in limited viral suppression in second-line treated patients. Therefore, for second-line ART patients with lower baseline CD_4_^+^ T lymphocyte counts, comprehensive drug resistance testing should be implemented, and the frequency of CD_4_^+^ T lymphocyte count and viral load measurements should be increased to facilitate timely treatment regimen adjustments, ensuring viral suppression and immune recovery. Although previous studies have not found an association between transmission route and the risk of LLV among people living with HIV (25), the present study observed that participants who acquired HIV through same-sex transmission experienced a higher risk of LLV (i.e., higher hazard) during second-line ART than those infected through other routes. Sexual orientation itself is unlikely to have any direct biological effect on virological outcomes. Prior literature suggests that factors such as HIV-related internalized stigma, multiple sexual partnerships, psychological distress, and substance use may influence treatment adherence (26, 27); however, these social–behavioral variables were not measured in our study, and therefore may represent unmeasured potential confounders. Accordingly, this observed association should be interpreted with caution.

Previous studies have shown that INSTI-based regimens can effectively reduce the risk of LLV and VF, particularly in treatment-naïve populations, where they rapidly and potently suppress viral load (27–29). However, in the present study of second-line patients, the DTG + 3TC + TDF regimen was associated with a higher risk of LLV and VF. Because this study is a non-randomized retrospective cohort, the observed association may be attributable to indication confounding and residual baseline differences between groups. Although we adjusted for measurable confounders—including baseline viral load, CD_4_^+^ lymphocyte levels, and known RAMs—unmeasured factors, such as the breadth of prior resistance profiles, details of treatment history, and clinicians’ prescribing preferences, may still have introduced bias. Therefore, this association should be interpreted with caution. Our findings suggest that NRTI RAMs may represent the key biological mechanism underlying this phenomenon. Resistance-associated mutations accumulated after failure of first-line therapy may explain the higher risk of LLV and VF observed in the DTG + 3TC + TDF group. Notably, at the VF level, both M184V and K103N were significantly associated with an increased risk of virologic failure. M184V can markedly reduce susceptibility to 3TC (30), suggesting that the 3TC component of DTG + 3TC + TDF may have limited contribution in the second-line setting. Meanwhile, although K103N is not a direct susceptibility site for 3TC/TDF, its association with a higher risk of VF may reflect more complex prior treatment exposures and a greater burden of drug resistance. We hypothesize that the higher incidence of LLV and VF observed with DTG + 3TC + TDF may be related to a resistance background that is more common after first-line failure in this group and is more likely to sustain viral replication. In contrast, the inhibitory effect of the LPV/r regimen is mainly driven by protease inhibition and, in theory, is less dependent on the NRTI backbone (31–33). However, because this study did not perform phenotypic drug-susceptibility testing (e.g., phenotypic susceptibility and fold change for TDF) and did not validate phenotypic effects for specific RAMs (e.g., K65R), the mechanistic interpretation based on resistance-associated mutations should be interpreted with caution.

Previous studies have not found a direct association between hepatitis B co-infection or HCV seropositivity and LLV/VF (25). However, the present study observed that individuals with hepatitis B co-infection or HCV seropositivity had higher hazards of LLV and VF during second-line ART relative to people living with HIV without these comorbidities. This difference might be partly attributable to the potential for hepatitis B co-infection and HCV seropositivity to elevate HIV viral load and impair virological suppression (34–36). Such interactive effects may be negligible under first-line ART, given that first-line regimens usually produce robust HIV suppression. By contrast, patients switching to second-line ART frequently have incomplete immune restoration and suboptimal viral control before regimen switching. Together with the effects of hepatitis B co-infection and HCV seropositivity, these factors may collectively contribute to elevated hazards of LLV and VF. Notably, HCV seropositivity in this study was determined solely by anti-HCV antibody testing, without systematic HCV-RNA testing. Accordingly, we cannot differentiate active HCV infection from resolved HCV infection, and these findings should be interpreted with caution.

Specific resistance-associated mutation loci also affect the risk of LLV and VF in second-line ART patients. This study found that patients carrying the M184V resistance-associated mutation had a higher risk of LLV than those carrying other resistance-associated mutations, whereas patients with the K103N resistance-associated mutation had a higher risk of VF. Previous studies have indicated that M184V and K103N are more prevalent in LLV/VF populations and that a proportion of these cases progress to VF during follow-up (37). M184V primarily confers high-level resistance to lamivudine (3TC) and emtricitabine (FTC). It is generally considered that this mutation does not lead to an enhanced resistance to tenofovir disoproxil fumarate (TDF) and zidovudine (AZT), and may even improve susceptibility, potentially due to reduced viral fitness and alterations in the resistance pattern (30). K103N primarily mediates resistance to non-nucleoside reverse transcriptase inhibitors (NNRTIs), including efavirenz (EFV), nevirapine (NVP), and other NNRTI drugs (38). Therefore, resistance-associated mutations suggest a greater treatment failure burden and more complex resistance profiles, which make virologic suppression more difficult in second-line therapy. Even when such patients transition to second-line ART after failure of first-line ART, the reduced drug susceptibility conferred by these resistance-associated mutations continues to affect subsequent treatment outcomes in people living with HIV.

## 5 Limitations

This study has the following limitations. First, we excluded patients with poor adherence and those with concomitant interfering conditions or treatments, including active malignancies, long-term immunosuppressive therapy, uncontrolled opportunistic infections (e.g., tuberculosis), and pregnant or breastfeeding women. Because poor adherence is a strong predictor of LLV and VF, excluding this high-risk group may limit the external validity of our findings. Our results primarily apply to patients who achieve and maintain sustained virologic suppression after second-line ART and, therefore, should not be directly generalized to the excluded populations. In addition, our sample was drawn from southwestern Guizhou Province, China. The local epidemiologic characteristics (e.g., viral subtypes, transmission routes, and comorbidity burden) and the availability of antiviral treatment strategies and resources may differ from other regions; thus, caution is warranted when extrapolating the findings to different settings or healthcare systems. Second, as a retrospective observational cohort study, we could only adjust for measurable confounders and could not fully control for unmeasured confounding. Second-line regimens were selected by clinicians based on routine real-world practice rather than randomized allocation, which introduces potential confounding by indication and residual confounding. For example, patients receiving specific regimens (including DTG + 3TC + TDF) may differ in terms of the complexity of their prior treatment history, the breadth and severity of resistance profiles, the virologic disease severity at transition to second-line therapy, and clinicians’ prescribing preferences; however, these factors may not be sufficiently quantified or adjusted for in retrospective medical records, potentially influencing the observed association between second-line regimens and LLV/VF. Third, due to the constraints of retrospective medical record data, we were unable to assess medication adherence with high granularity. Residual differences in adherence may still have affected the study outcomes. Fourth, this study defined HCV seropositivity based on baseline anti-HCV antibody results, while systematic HCV-RNA testing was not available in this cohort. Therefore, we could not distinguish individuals with active chronic HCV infection from those with resolved past HCV infection. This lack of virological verification may limit the precision and interpretation of the observed association between HCV seropositivity and LLV/VF.

## 6 Conclusion

This study, based on a cohort of HIV patients in Southwest China, investigated factors associated with the occurrence of LLV and VF in second-line ART patients. The results indicate that baseline CD_4_^+^ T lymphocyte count, HIV infection through homosexual behavior, the use of DTG + 3TC + TDF as a second-line treatment regimen, hepatitis B co-infection or HCV seropositivity, and carrying specific drug resistance mutations may increase the risk of LLV and VF. Therefore, it is recommended that HIV-infected individuals should initiate ART as early as possible. During second-line treatment, it is crucial to strengthen drug resistance testing and dynamic monitoring of CD_4_^+^ T lymphocyte counts to allow for timely adjustment of treatment regimens, improve patient prognosis, and promote the achievement of the “Undetectable = Untransmittable” (U=U) goal.

## Data Availability

The raw data supporting the conclusions of this article will be made available by the authors, without undue reservation.
